# Induction of Robust Future Liver Remnant Hypertrophy Before Hepatectomy With a Modified Liver Venous Deprivation Technique Using a Trans-venous Access for Hepatic Vein Embolization

**DOI:** 10.3389/fradi.2021.736056

**Published:** 2021-11-30

**Authors:** Nils Degrauwe, Rafael Duran, Emmanuel Melloul, Nermin Halkic, Nicolas Demartines, Alban Denys

**Affiliations:** ^1^Centre Hospitalier Universitaire Vaudois (CHUV), Lausanne, Switzerland; ^2^Department of Diagnostic Radiology and Interventional Radiology, Lausanne University Hospital (CHUV), Lausanne, Switzerland

**Keywords:** liver, embolization, Klatskin, portal embolization, liver resection

## Abstract

**Purpose:** Hepatic and/or portal vein embolization are performed before hepatectomy for patients with insufficient future liver remnant and usually achieved with a trans-hepatic approach. The aim of the present study is to describe a modified trans-venous liver venous deprivation technique (mLVD), avoiding the potential risks and limitations of a percutaneous approach to hepatic vein embolization, and to assess the safety, efficacy, and surgical outcome after mLVD.

**Materials and Methods:** Retrospective single-center institutional review board-approved study. From March 2016 to June 2019, consecutive oncologic patients with combined portal and hepatic vein embolization were included. CT volumetric analysis was performed before and after mLVD to assess liver hypertrophy. Complications related to mLVD and surgical outcome were obtained from medical records.

**Results:** Thirty patients (62.7 ± 14.5 years old, 20 men) with liver metastasis (60%) or primary liver cancer (40%) underwent mLVD. Twenty-one patients (70%) had hepatic vein anatomic variants. Technical success of mLVD was 100%. Four patients had complications (three minor and one major). FLR hypertrophy was 64.2% ± 51.3% (mean ± SD). Twenty-four patients (80%) underwent the planned hepatectomy and no surgery was canceled as a consequence of mLVD complications or insufficient hypertrophy. Fifty percent of patients (12/24) had no or mild complications after surgery (Clavien-Dindo 0–II), and 45.8% (11/24) had more serious complications (Clavien-Dindo III–IV). Thirty-day mortality was 4.2% (1/24).

**Conclusion:** mLVD is an effective method to induce FLR hypertrophy. This technique is applicable in a wide range of oncologic situations and in patients with complex right liver vein anatomy.

## Introduction

Surgical resection is the principal curative option in patients with primary hepatic tumors and liver metastases. Unfortunately, resectability may be limited by volume and function of the liver remnant after surgery [known as the future liver remnant (FLR)] (1). Against this limitation, strategies to increase FLR rapidly before surgery were developed to prevent postoperative liver insufficiency. Portal vein embolization (PVE) was the first technique developed in the late 1980s (2) and became widely used because it was highly reliable and allowed liver hypertrophy with low morbidity (3). A major concern in clinical practice was the risk of insufficient hypertrophy and increased delay between PVE and resection, resulting in resectability rates between 60 and 85% only, after a delay of 4–6 weeks (4, 5). More invasive surgical strategies have also been developed like ALPPS: Associating Liver Partition and Portal vein Ligation for Staged hepatectomy (6). Despite excellent results for hypertrophy, complications and mortality rate were too high and some questions about the functional quality of the hypertrophied liver limited the initial enthusiasm for this approach (7).

Due to the respective limitations of PVE and ALPPS, an interesting technical improvement has been developed: liver venous deprivation (LVD). LVD combines percutaneous portal and hepatic vein embolization during the same procedure (8, 9). Although no prospective randomized comparison between techniques was performed, LVD resulted in robust and fast hypertrophy compared to PVE, particularly when both the right and middle hepatic veins are occluded (9). In the initial LVD reports, hepatic veins were embolized through a 7-9F percutaneous access using a plug located 2 cm upstream from the inferior vena cava (IVC). The branches were additionally occluded with glue (8). The percutaneous LVD technique has several limitations. Firstly, hepatic vein flow is stopped after deployment of the plug and glue injection is subsequently performed with risk of migration. Indeed, as observed by Guiu and colleagues, collateral between hepatic veins can be immediately visible and there is a risk of glue passage through collaterals to adjacent veins that limits its use to high experience operators (8). Secondly, multiple large diameter transhepatic accesses (at least 7F sheaths) are needed in cases of multiple hepatic veins (either right and middle and/or right hepatic vein anatomic variants). Furthermore, if right inferior hepatic veins are easy to access from a percutaneous route, accessory branches from segment VII or VIII branching in the IVC or in the distal part of the right hepatic vein are not easily punctured as noted by Guiu in its first report (8). Of note, this technique should not be used in the presence of Klatskin tumors associated with biliary dilatation (due to the risk of biliary leak along the percutaneous puncture tract). To overcome the limitations of the percutaneous technique, we developed a modified liver venous deprivation (mLVD) technique. Our bet was that the trans-jugular (or trans-femoral) approach could embolize multiple hepatic veins and variants mostly with a single peripheral venous puncture and that it would be applicable in patients with complex oncologic situations (biliary dilatation or large hepatic tumors).

The aim of the present study was to describe the mLVD technique and to assess the safety, efficacy, and surgical outcome of/after the procedure.

## Materials and Methods

### Patients and Design

This is an IRB-approved (registered under record number 2019-00444), single-center, retrospective study. All 30 consecutive patients who underwent liver venous deprivation for oncologic conditions between March 2016 and June 2019 were included. All patients provided written informed consent before the procedure. All patients were discussed at the liver tumor board meeting, including medical oncologists, hepatobiliary surgeons, and pathologists, as well as diagnostic and interventional radiologists. An insufficient FLR was defined as baseline FLR < 25% of the total liver volume in patients with healthy liver. In patients with Klatskin tumors and hepatocellular carcinoma, or after multiple cycles of neoadjuvant chemotherapy, an insufficient FLR was defined as baseline FLR <40% of the total liver volume (10). Patients' age, gender, and underlying liver disease were registered.

### Treatment Planning

**Figure 1:**
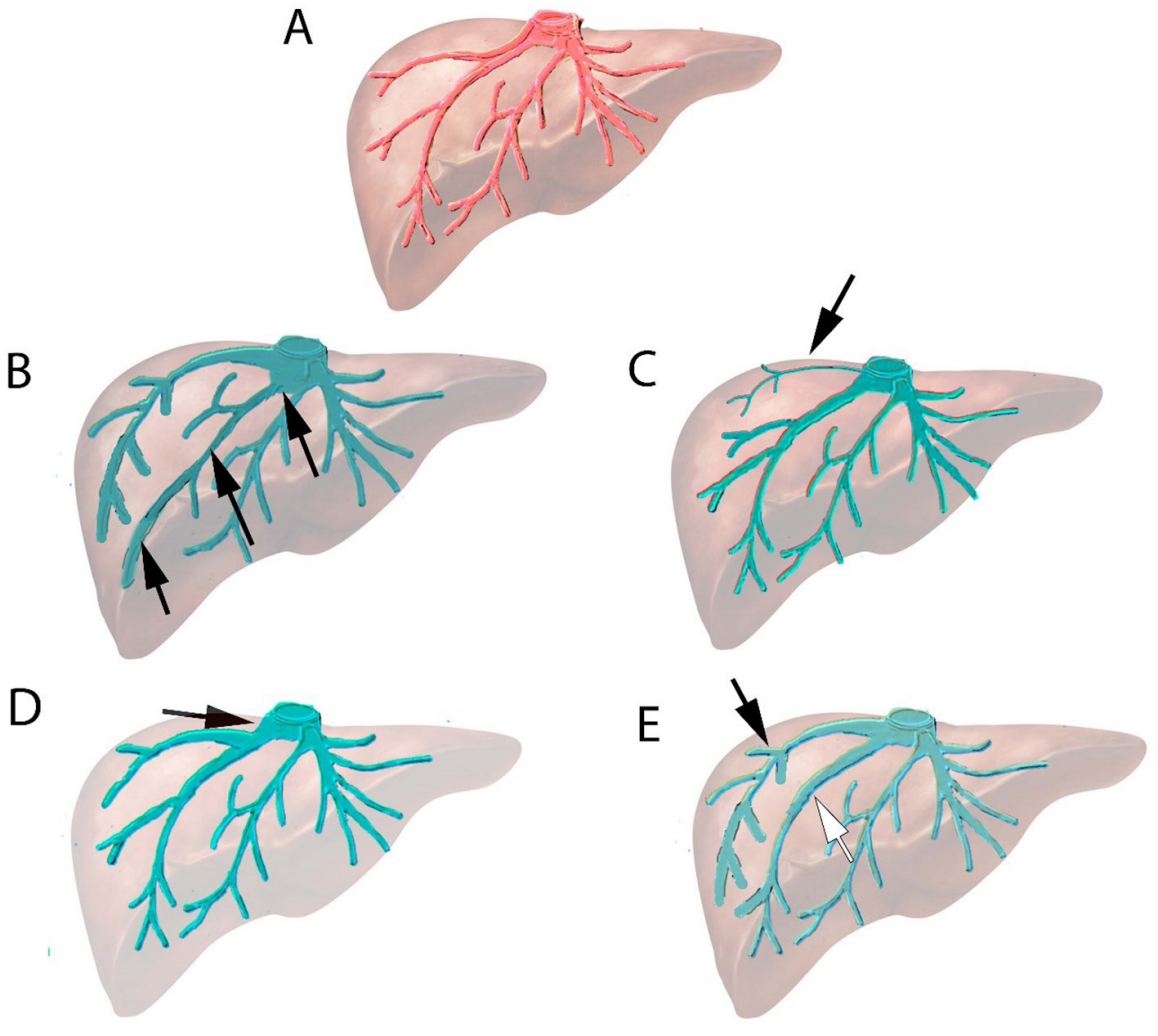
Normal anatomy and variants in the right hepatic vein system. **(A)** Normal anatomy of hepatic veins. **(B)** Anatomical variant: Large inferior hepatic vein branching directly to inferior vena cava (IVC) (black arrow). **(C)** Anatomical variant: Vein of segment VII (arrow) branching directly to IVC. **(D)** Anatomical variant: Segmental vein connecting to the last centimeter (arrow) of hepatocaval confluence. **(E)** Anatomical variant: Two right hepatic veins of similar size (duplication of right hepatic veins) merging just upstream of hepatocaval confluence (white and black arrow).

Imaging (abdominal CT-scan and/or liver MRI) was reviewed before the embolization procedure to evaluate tumor size, location, and vascular anatomy. The anatomy of the hepatic veins was analyzed and anatomical variabilities were classified following the criteria that have been previously reported in the following way: (1) a large inferior right accessory vein draining directly into the inferior vena cava (IVC); (2) a small accessory vein of segment VII draining directly into the IVC; (3) branching of segmental veins very close to the hepatocaval confluence (usually in the last centimeter); and (4) duplication of the right hepatic vein (Figure 1). These are the variants of the hepatic veins in the right hemi-liver described in large series analyzed by computed tomography (11) or in cadaveric study (12).

### MLVD Technique and Technical Outcome

Treatment planning included a review of the patient's liver anatomy on the pre-intervention cross-sectional imaging. The patient was placed in supine position under general anesthesia. The right side of the neck and the upper abdomen were prepped. The first step of the procedure was selective PVE as described elsewhere (13, 14). Briefly, a small peripheral portal vein of segment III was punctured under ultrasonographic control. A 5F introducer sheath was placed according to the Seldinger technique, and the portal trunk was catheterized using a Berenstein catheter advanced over a stiff guide wire. After having performed a baseline portography, right portal branches were selectively catheterized and embolized using a mixture of n-butyl-cyanoacrylate (Braun) and lipiodol (Guerbet) (ratio 1:3 or 1:5). Portal vein pressures were measured before PVE and after liver venous deprivation (end of the procedure). Right portal vein embolization was performed before hepatic vein embolization, because hepatic vein embolization reduces portal flow in the embolized segment making portal vein embolization more at risk for non-target embolization as demonstrated in two animal studies (15, 16). Although both a left or right portal vein approach can be used for right PVE, we prefer using a contralateral approach, which makes the procedure technically easier (e.g., avoiding catheter kinking), and is safer in case of right liver biliary tree dilatation as observed in Klatskin tumors (17).

**Figure 2:**
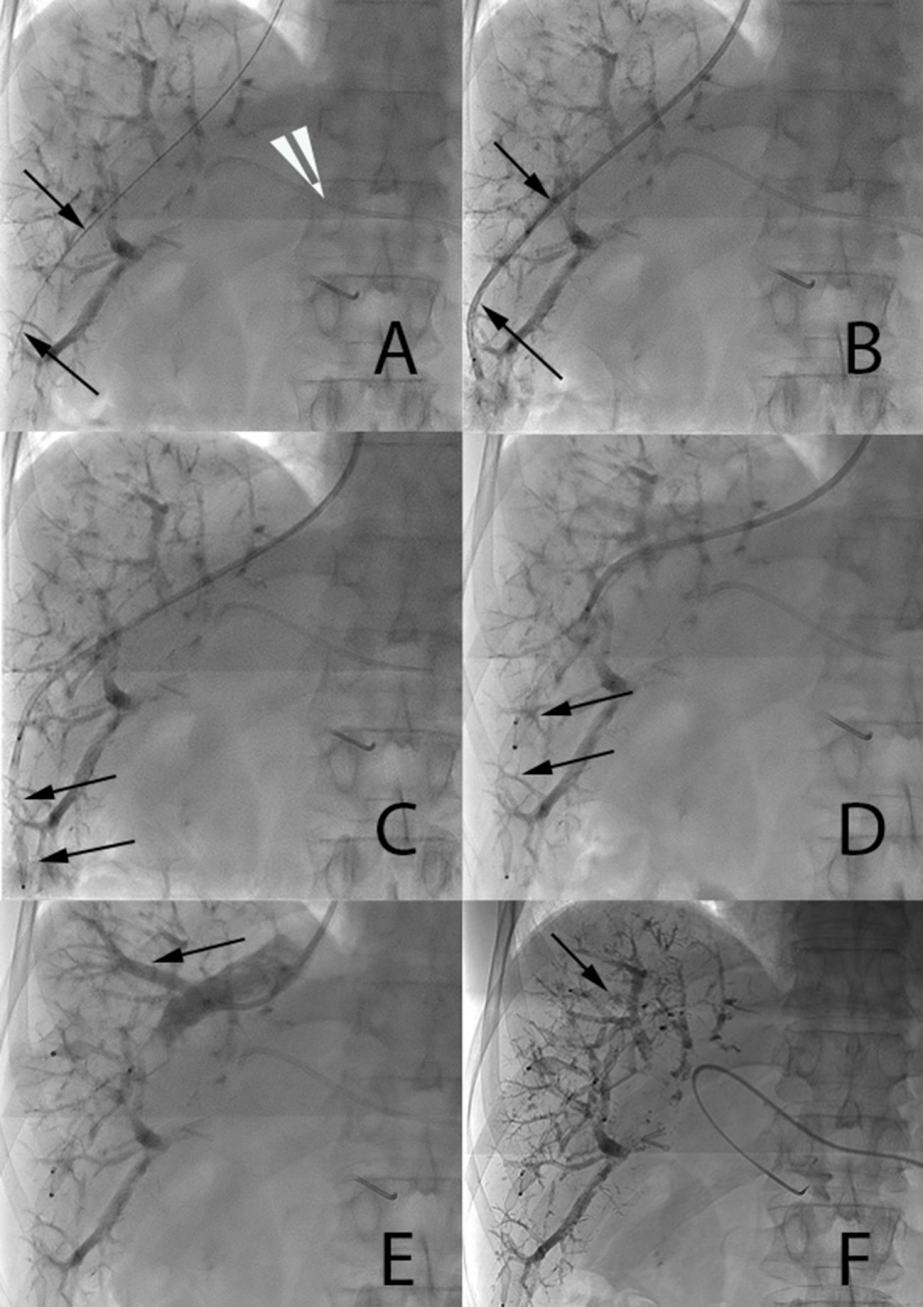
Main steps of endovascular hepatic vein occlusion. **(A,B)** A 0.035-inch stiff guide wire (arrow 2a) and a 7F catheter (arrow 2b) are placed distally in the right hepatic vein. 5F Berenstein catheter visible in the left portal vein (white arrow tip). **(C,D)** A first 12-mm Amplatzer vascular plug II and then a 16-mm one are progressively deployed under fluoroscopic control one along the other in order to ensure stability (arrows). **(E)** Injection of contrast media through the catheter demonstrates a large segmental vein (arrow). **(F)** Occlusion of this large segmental vein with an additional 10-mm plug (arrow).

For hepatic vein embolization, the right internal jugular vein was located and punctured under ultrasound guidance (Figure 2). A 9F 65-cm angulated introducer sheath (Cook Medical) was advanced to the distal part of the right hepatic vein and the hepatic veins and their variants were catheterized based on their locations identified on pre-procedural imaging. When needed, for more distal catheterization, an 8F MACH 1 guiding catheter may be used (Boston Scientific). Each of the branches of the right hepatic vein (and if needed, the middle hepatic vein as well) was then catheterized and occluded using an Amplatzer Vascular Plug II (Abbott) that is at least 50% larger than the diameter of the vein. After the first plug released peripherally into the small distal branches, the next was placed, whenever possible, to the distal end of the first one to achieve an overlap of the plugs for optimized embolization and stability. Several vascular plugs were thus subsequently placed from the liver periphery to centrally toward the IVC. The last 2–3 cm of the hepatic vein close to the IVC were not embolized in order to ease dissection and clamping close to the IVC during surgery. Femoral access was occasionally used in case of difficult catheterization (due to unfavorable angulation) of hepatic or an accessory vein. In case of planned extended right hepatectomy (liver segments IV to VIII), middle vein occlusion was performed using the same technique, with the aim of improving FLR hypertrophy.

The duration of mLVD procedure was recorded. Vascular access type (jugular, femoral, or both), the number of embolized veins, and the number of plugs used were recorded. Technical success and mLVD-related complications (e.g., migration of occlusion device, bleeding, or complications at the puncture site) were monitored and classified according to the scale of the Society of Interventional Radiology (18). Portal venous pressure was measured before and after mLVD.

### Volumetric Calculations

Liver volumetric analysis was performed on a PACS workstation (Carestream Vue PACS, Carestream Health). The FLR, whole liver volume, and tumor volume(s) were segmented manually and calculated before mLVD and before surgery. FLR is defined as the liver volume that will remain after the hepatectomy, with its central limit along the planned future surgical gross section. The FLR ratio was defined as FLR divided by (total liver volume – tumoral volume). FLR hypertrophy was defined as [(FLR after embolization – FLR before)/FLR before)^*^100] (9, 13, 19).

The estimated FLR ratio (eFLR) represents the FLR divided by the estimated total liver volume calculated according to the following formula: total liver volume (cm^3^) = 1,267.28 × body surface area (BSA) (m^2^) – 794.41. It has been shown that liver volume calculation based on BSA is precise and reliable (19). It is not influenced by parameters such as biliary dilatation or vascular obstruction that may modify “non-functional” volume of compromised liver. Therefore, eFLR is also reported in this study as an alternative method for total liver volume calculation.

### Surgical Procedure

Hepatectomy was performed according to standard surgical techniques described elsewhere (20, 21). The following data were obtained: resectability, perioperative blood loss, and surgical morbidity based on the Clavien classification (22).

### Statistical Considerations

Unless otherwise stated, continuous variables were presented as means ± standard deviations (SD) and binary variables were presented as *N* and percentages. Paired *t-*test was used for hypertrophy significance calculation before and after mLVD. Statistical significance was defined as *p* < 0.05. Statistical analysis was performed using GraphPadPrism 8 (GraphPad Software, La Jolla, CA, USA).

## Results

### Patients

Thirty patients underwent mLVD. Sixty-seven percent of patients (20/30) were men, and 33% (10/30) were women, mean age 62.7 ± 14.5 years old. Eighteen patients (60%) had colorectal liver metastases, 10 (33%) had hilar cholangiocarcinoma, and 2 (7%) had hepatocellular carcinoma.

### MLVD and Hepatic Venous Anatomy

Fourteen (47%) patients had a right accessory vein draining directly into the IVC, which constitutes the most frequent variation observed (large inferior accessory vein or small segment VII vein connecting directly to IVC, described in Figures 1B,C, respectively). Four patients (13.3%) had branching of a large segmental vein into the right hepatic vein upstream from the hepatocaval confluence (last centimeter) (Figure 1D). Three patients (10%) had a duplication of the right hepatic vein (Figure 1E).

### MLVD Data and Safety

**Figure 3:**
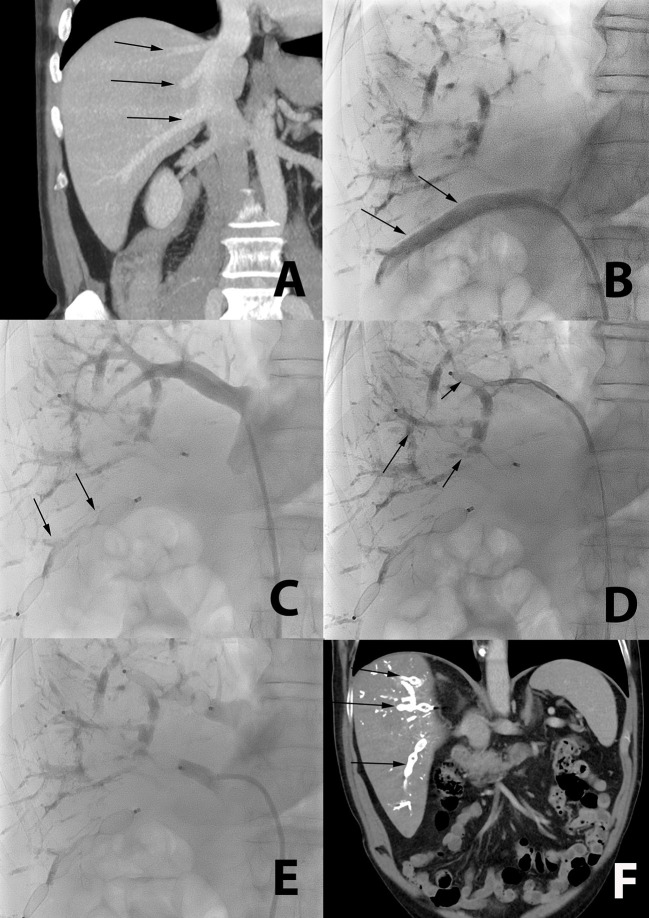
Occlusion of multiple accessory veins using a single femoral access in a 72-year-old patient with liver metastasis from colorectal cancer. **(A)** CT coronal reformation of a patient with large segmental inferior right hepatic vein (lower arrow), and two smaller segmental veins (middle and upper arrow), all draining directly in the inferior vena cava. **(B)** Catheterization (arrow) of the large segmental inferior hepatic vein using a femoral access. **(C)** Plugs are deployed in this large segmental inferior vein (arrow). **(D,E)** The two other segmental veins are subsequently catheterized and occluded with plugs (arrow). **(F)** Post-mLVD CT coronal reformation showing occlusion of all segmental veins with the plugs (arrows).

The total mLVD procedure lasted 101 ± 41 min. Thirteen patients had two different occluded veins, six patients had three occluded veins (Figure 3), and one patient had four occluded veins with the mLVD technique. Dual jugular and femoral access was necessary in 3 out of 30 patients (10%).

The right hepatic vein was occluded in all 30 patients and 4 patients had middle vein occlusion. The technical success was 100%. One patient experienced a major complication, active hemorrhage (originating from an internal thoracic artery branch), which was a consequence of percutaneous puncture for PVE (Grade C complication according to the Society of Interventional Radiology (SIR) classification system) (18). This patient required selective embolization. Three patients had minor complications (Grade A according to SIR): one patient had a cytolysis with >10-fold increase in liver enzymes and spontaneous return to baseline within 10 days. One patient had an allergic reaction to iodine-based contrast, with mild skin rash, and one patient had a small subcapsular hepatic hematoma without active hemorrhage. No hepatectomy was canceled or delayed because of mLVD complication. No migration of the occlusion plugs into the heart and/or pulmonary arteries was observed in the 30 mLVD procedures (average of five plugs per procedure; range, three to nine). Right portal vein embolization was successful in all patients (two to four vials of cyanoacrylate n-butyle-2). All branches of the right portal vein system were occluded (except segment IV). No residual flow was observed on final portography. The mean change in portal pressure after mLVD was + 2.3 ± 1.6 mmHg (data available in 15 patients). Per-procedural biliary drainage was performed in 6 out of the 10 cholangiocarcinoma patients, while 2 had pre-procedural endoscopic retrograde drainage.

### FLR Hypertrophy After MLVD

Liver volumes could be determined in 29/30 patients (one patient was subsequently treated in another institution abroad and lost to follow-up). Liver volumes were determined, with a mean of 28.6 ± 18.3 days after embolization. Before embolization, the FLR ratio was 30.6% ± 8.2%, and after embolization, the FLR ratio was 41.7% ± 8.5% (*p* < 0.0001). FLR hypertrophy was 64.2% ± 51.3%. Before embolization, the eFLR ratio was 34.5% ± 11.1%. After embolization, eFLR ratio was 53.2% ± 15.3% (*p* < 0.0001). No surgery was canceled because of insufficient FLR hypertrophy. Data are summarized in Table 1.

**Table 1:** Summary of hypertrophy results.

	**Before mLVD**	**After mLVD**	***p*-value**
Mean FLR cc	524 (±180)	806 (±218)	<0.0001
FLR ratio (%)	30,6 (±8.2)	41.7 (±8.5)	<0.0001
eFLR	34,5 (±11.1)	53,2 (±15.3)	<0.0001
FLR hypertrophy		64.2 %	

*mLVD, modified liver venous deprivation; FLR, future liver remnant; eFLR, estimated future liver remnant. Values are represented ± standard deviation in the parentheses. For details about calculations and corresponding formulas please see material and methods section*.

### Outcome of Surgery

Hepatectomy was performed 43.4 ± 26 days after mLVD. The mean length of hospitalization after hepatectomy was 18.8 days ± 13.6 days. There were no surgical complications secondary to mLVD, such as adhesions, difficulty for clamping, or ligating hepatic veins during hepatectomy. Blood loss was 1,019 ± 466 ml. No patients suffered from post-operative liver insufficiency.

Twenty-four out of 30 patients (80%) underwent the planned hepatectomy. Three (10%) patients were no longer eligible for hepatectomy after mLVD because of disease progression observed on imaging (*n* = 1) or due to medical complications unrelated to the mLVD procedure (congestive heart failure, stroke, and pulmonary embolism, *n* = 2). Two patients went to surgery, but unresectable progressive disease was discovered during laparotomy and hepatectomy was canceled. One patient was lost to follow-up because of subsequent treatment in another country.

General complications following hepatectomy were recorded and classified using the Clavien–Dindo classification system. Eight out of 24 (33.3%) patients had no complications, 3/24 (12.5%) had grade I complications (1 patient with transient tachycardia without need for pharmacological intervention, 1 patient with peripheral edema and ascites treated with diuretics, and 1 patient with subcutaneous abscess that was treated with bedside incision), 2/24 (8.3%) had grade II complications (anemia treated with blood transfusion; post-operative infection treated with antibiotic administration), 2/24 (8.3%) had grade IIIa complications (seromas that required drainage), 6/24 (25%) had grade IIIb complications (5 patients had biliary leak with re-intervention or drainage under general anesthesia and 1 patient had a gastric ulcer who required endoscopy under general anesthesia), and 2/24 (8.3%) had grade IV complications (patients with potentially life-threatening bleeding that required admission to the ICU). One patient died 24 days after surgery from multi-organ failure and uncontrolled bleeding (grade V, 30-day mortality 4.2%).

## Discussion

In the present study, we performed liver venous deprivation by using a transvenous (jugular or jugular and femoral) approach for hepatic vein embolization, in 30 consecutive patients with various primary or metastatic liver oncologic diseases. We observed that 70% of patients had anatomical variations in the right liver vein system, all of which could be catheterized and occluded. Taking advantage of the “endovascular” approach, mLVD was performed even in the presence of right liver biliary tree dilatation, as observed in hilar cholangiocarcinoma/Klatskin tumors, which represent 30% of patients treated in this study. No biliary leak secondary to mLVD occurred in this study. Only one major post-procedural complication (hemorrhage) occurred and was related to the PVE part of the procedure, highlighting the risk of percutaneous approaches to the liver in general, even with 5F diameter accesses such as the ones used for portal vein embolization. In our study, mLVD induced robust hypertrophy in all patients. Thus, no intervention was canceled or delayed because of insufficient FLR hypertrophy neither because of complications related to the procedure.

FLR hypertrophy was 64.2% with mLVD, and thus comparable to other LVD series, respectively 52.6 and 53.4% in the reports by Guiu et al. (8, 9). Faster or increased FLR hypertrophy of LVD compared to PVE is supported by retrospective evidence, with similar surgical outcome (23, 24). Although no peri-procedural complications are described in the initial LVD reports, direct comparison of a percutaneous and endovascular approach is difficult given the small number of patients in the LVD series (7 and 10 patients) and the relatively low occurrence of complications in general (8, 9). A more recent study compared peri-operative outcome after PVE (16 patients) or LVD (13 patients) and showed similar mortality and morbidity with both techniques but did not detail potential complications related to LVD. Ten out of 13 patients in the LVD group had colorectal liver metastasis, 3 out of 10 hepatocellular carcinoma, while no Klatskin tumors were treated with this technique (24). An ongoing prospective trial is comparing PVE with LVD and will provide more clues regarding hypertrophy and complications of both techniques (25). In the currently available literature, comparison among techniques is difficult because it is likely that patients are selected for a given procedure depending on their hepatic venous anatomy or risk of complications (e.g., major biliary tree dilatation). To the best of our knowledge, no data originating from a randomized controlled trial comparing PVE with LVD are currently available.

The present study highlights the possibility to occlude multiple hepatic and accessory veins mostly with a single jugular punction. This is especially relevant in case of anatomical variants of hepatic vein in the right liver described in up to 50% of patients in the literature and found in 70% of patients in the present study. Particularly relevant variants from a technical point of view include direct drainage into the IVC (inferior hepatic vein) or proximal connection of a segmental vein in the last 2 cm of the hepatocaval confluence, all of which can be treated with mLVD (11, 12, 26). It is possible that patients with complex right liver vein anatomy were not eligible for the classical LVD technique because it would have required too many percutaneous punctures. Indeed, after anatomical analysis and classification, it appears that only 33% of patients would have required a single percutaneous puncture for hepatic vein embolization if a percutaneous approach would have been used in the population of the present study. Data regarding number of punctures required for LVD and anatomical variants are limited in the currently available literature. Of note, Guiu et al. mention in their initial LVD report that they could not occlude two accessory veins of segment VIII due to technical limitations (8). The consequence of a percutaneous approach may thus be to overlook some variant draining veins or technical inaccessibility due to unfavorable angulation. Moreover, venous communications are described between right liver segments in 27% of cases in a post-mortem analysis, suggesting a possible risk of glue migration from one venous territory to another with percutaneous LVD technique (12). Incomplete occlusion of accessory veins and flow through collaterals may negatively impact FLR hypertrophy, as suggested in a pre-clinical study (15). However, superiority of mLVD over LVD in occlusion of accessory veins and prevention of flow through collaterals remains to be studied. Non-target hepatic vein embolization did not occur in our study, because the mLVD technique uses vascular plugs, which are deployed in a large part of the target vein, and are thus more likely to cover collateral veins. Should the chosen size of the vascular plug be inadequate (too small with migration risk), it can be easily and safely removed and changed for the adequate size plug. In the present experience, by oversizing the plug by 50% of the vein size, embolization of the vein was optimal and no plug did migrate. Another advantage of the endo-venous approach is that it works “against” the venous flow, with a large catheter downstream from the plug. In case of insufficient size with potential migration, this is safer than percutaneous approach upstream from the plug and the flow. Moreover, plugs can be “overlapped” for optimized embolization and stability. An additional important consideration is that liver tumoral invasion can induce upstream dilatation of bile ducts, which is a relative contraindication to percutaneous liver punction. Repeated percutaneous punctures with large-bore access may be required to deploy plugs in classical percutaneous LVD technique. Further work is required to confirm an advantage of mLVD over LVD in terms of peri-procedural complications and surgical outcome. Finally, in the present study, 80% of patients underwent the planned hepatectomy after mLVD, which is in the upper range compared to published series, especially considering the high percentage of patients with cholangiocarcinoma/Klatskin tumors (4, 5, 27, 28).

The present study has several limitations including its retrospective design and a heterogeneous group of patients with various underlying oncologic diseases. In addition, liver volume calculation was not systematically carried out at the same time point after the procedure. Although this could influence FLR hypertrophy values, the ideal time point for FLR hypertrophy has not been defined yet, and this range is the result of a retrospective unselected population and thus “real-life” clinical practice. In addition, it seems that most of the hypertrophy is obtained at 7 days and then stagnates (9). Although no patient suffered from liver insufficiency after hepatectomy, liver function was not assessed systematically using Tc-99m mebrofenin scintigraphy in the present study and constitutes a limitation. Available retrospective evidence suggests that LVD induces not only greater FLR hypertrophy but also increased FLR function compared to PVE alone (29). One technical disadvantage of mLVD is that several plugs are needed, which increases the costs of the procedure. Radiation burden is also increased, given the extended procedural time. However, precise values cannot be provided since radiation burden is available for the total procedure and the amount attributable to hepatic vein occlusion can therefore not be measured accurately in our retrospective study. Besides liver volume, other oncological critical factors will have to be evaluated in the future: complications of the various embolization procedures/techniques and their association with delayed or canceled surgeries; time to hypertrophy to prevent the development of extra-hepatic disease or uncontrolled local growth before surgery; and “quality” of the hypertrophy and the effect on liver function and outcome (30, 31).

In conclusion, the present work describes an alternative “endovascular” method to hepatic vein occlusion in LVD, which was safe and induced robust liver hypertrophy, comparable to that of the classical percutaneous LVD technique. Importantly, it was feasible in a wide range of clinical situations, including patients with complex right liver vein anatomy or right liver biliary tree dilatation.

## Data Availability Statement

The raw data supporting the conclusions of this article will be made available by the authors, without undue reservation.

## Ethics Statement

The studies involving human participants were reviewed and approved by CER-VD (Commission Éthique du canton de Vaud). The patients/participants provided their written informed consent to participate in this study.

## Author Contributions

ND and AD wrote the manuscript and performed study design and data analysis. NH, RD, and EM performed study design and reviewed manuscript. All authors contributed to the article and approved the submitted version.

## Conflict of Interest

The authors declare that the research was conducted in the absence of any commercial or financial relationships that could be construed as a potential conflict of interest.

## Publisher's Note

All claims expressed in this article are solely those of the authors and do not necessarily represent those of their affiliated organizations, or those of the publisher, the editors and the reviewers. Any product that may be evaluated in this article, or claim that may be made by its manufacturer, is not guaranteed or endorsed by the publisher.
